# What Do United States Adolescents Eat? Food Group Consumption Patterns and Dietary Diversity from a Decade of Nationally Representative Data

**DOI:** 10.1016/j.cdnut.2023.101968

**Published:** 2023-06-29

**Authors:** Mica Jenkins, Maria Elena D. Jefferds, Nancy J. Aburto, Usha Ramakrishnan, Reynaldo Martorell, O. Yaw Addo

**Affiliations:** 1 International Micronutrient Malnutrition Prevention and Control (IMMPaCt) Program, Nutrition Branch, Division of Nutrition, Physical Activity, and Obesity, National Center for Chronic Disease Prevention and Health Promotion, Centers for Disease Control and Prevention (CDC), Atlanta, GA, United States; 2 Nutrition and Health Sciences Doctoral Program, Laney Graduate School, Emory University, Atlanta, GA, United States; 3 Food and Nutrition Division, Food and Agriculture Organization of the United Nations, Rome, Italy; 4 Hubert Department of Global Health, Rollins School of Public Health, Emory University, Atlanta, GA, United States

**Keywords:** United States adolescents, dietary diversity, food group consumption patterns

## Abstract

**Background:**

Although the importance of adolescent nutrition has gained attention in the global nutrition community, there is a gap in research focused on adolescent dietary diversity and food group consumption.

**Objectives:**

This study aimed to characterize population-level food group consumption patterns and quantify the extent of dietary diversity among United States adolescents using a large nationally representative sample of adolescents aged 10–19 y.

**Methods:**

We used 24-h dietary recall data from the National Health and Nutrition Examination Survey (NHANES) from 2007 to 2018 to construct the 10 food groups comprising the minimum dietary diversity for women (MDD-W) indicator and estimated the prevalence of intake of each food group. A composite metric adolescent dietary diversity score (ADDS) was derived for each adolescent where 1 point was awarded per food group. Both population scores and the distribution of individual scores were estimated. Differences in proportions of food groups consumed across sociodemographic categories were tested using the Rao–Scott χ^2^ test, and pairwise comparisons were expressed as population prevalence differences and prevalence ratios.

**Results:**

Food group consumption patterns were very similar across 2 d of dietary recall but varied significantly by sex, race/ethnicity, and income status. The food groups with the highest prevalence of consumption were grains, white, roots, and tubers (∼99%), milk products (∼92%), and meat, poultry, and fish (∼85%), whereas <15% of adolescents consumed key micronutrient-dense foods, such as vitamin A–rich fruits and vegetables and dark green vegetables. The mean ADDS was 4.69, with modest variation across strata.

**Conclusions:**

On average, United States youth consumed fewer than 5 food groups on a given day. The lack of dietary variety and relatively low prevalence of consumption of several micronutrient-rich plant-based foods could pose a risk for adolescents’ ability to achieve micronutrient adequacy in the United States.

## Introduction

Since 2015, the Dietary Guidelines for Americans have emphasized the importance of healthy eating patterns, recognizing that individual nutrients, foods, and food groups are not consumed in isolation but rather in various combinations over time [1]. The 2020 guidelines expanded the focus on healthy eating patterns to all stages of life, recognizing that patterns established early in life may have important implications for long-term health and chronic disease prevention [2]. The guidelines define a healthy eating pattern as one that “consists of nutrient-dense forms of foods and beverages across all food groups, in recommended amounts, and within calorie limits.” This definition inherently embraces the importance of dietary diversity, which research has shown is a proxy for micronutrient adequacy and associated with child nutritional status [[3], [4], [5], [6], [7], [8]].

The importance of nutrition in adolescence has steadily gained attention in the global nutrition community, spurring important research and publications, such as the 2016 and 2021 Lancet Series on adolescent health and nutrition [[9], [10], [11], [12], [13]]. This work has raised a call to action for researchers and program implementers to increase focus on adolescent nutrition with an emphasis on dietary diversity [14]. Simultaneously, the global nutrition transition has notably affected adolescent dietary behavior; for instance, a 2017 study of adolescent dietary intake across 4 developing countries (Ethiopia, India, Peru, and Vietnam) found significant changes over time, such as an increase in the prevalence of consumption of eggs, dairy, meat, fruits and vegetables, and added sugar among some cohorts [15].

Since the 1940s, the United States has enacted various state-level and national-level policies related to food fortification and enrichment that positively affected nutrient availability in the food system and intake by the United States population [[16], [17], [18]]. However, such policies are not universal and are limited to specific foods and nutrients. Recent studies have found high prevalence of inadequate intake of key nutrients among United States children [19,20] and adults [21,22] and high risk of multiple micronutrient deficiencies in both children and adults [23]. Given the complexity and cost of enforcing mandatory nationwide food fortification measures, the promotion of dietary diversity as a means of achieving micronutrient adequacy can be a cost-effective solution [24].

To date, several studies have characterized United States adolescent dietary quality using scores specifically intended for the United States population, such as the AHA’s continuous diet scores, the Healthy Eating Index, and Whole Plant Foods Density [25,26]. However, to our knowledge, none have used indicators with international relevance to characterize dietary diversity among adolescents in the United States population, such as individual dietary diversity score [27], women’s dietary diversity score [27], or minimum dietary diversity for women (MDD-W) [28]. These food group–based indicators are especially useful in contexts where regular collection of 24-h dietary recall data is not feasible due to limited resources, both technical and financial. In particular, MDD-W is a dichotomous indicator of dietary diversity in women of reproductive age, which is based on the consumption of 10 food groups and can be used as a proxy for higher micronutrient adequacy. Using the MDD-W food groups to describe dietary diversity among United States adolescents can enable comparisons between the United States and other country populations and improve knowledge of adolescent dietary patterns. This information can inform the design of adolescent health and nutrition programs and policies, particularly those aiming to alleviate micronutrient deficiencies.

The purpose of this study was to characterize food group consumption patterns and quantify the extent of dietary diversity among United States youth using a large nationally representative sample of adolescents aged 10–19 y. By aggregating 24-h dietary recall data into the 10 MDD-W food groups, we aimed to explore the usefulness of this simple indicator for describing dietary diversity among United States adolescents. In the future, such a tool could be used as an efficient and inexpensive alternative to in-depth 24-h dietary recall in some settings.

## Methods

### Data sources and population

We used data from six 2-y consecutive cycles (2007–2018) of the continuous NHANES to achieve a large sample required for our research purpose. NHANES is a surveillance system of NCHS, combining interviews and physical examinations to produce health information for the United States [29]. Each year, the survey examines a nationally representative sample of ∼5000 people across the country. NHANES field teams comprise physicians, medical and health technicians, and dietary and health interviewers, many of whom are fluent in both Spanish and English. Health interviews are conducted in respondents’ homes, whereas health measurements are performed in mobile examination centers.

### Dietary intake module

The dietary interview component of NHANES, known as What We Eat in America, is conducted in partnership with the USDA and the United States Department of Health and Human Services using validated data collection methods [30]. Two days of 24-h dietary recall data are collected for each participant by trained dietary interviewers. The first day of data is collected in a private room in the mobile examination centers and the second day is collected by telephone 3–10 d later. Interviews with children aged 9–11 y involve the assistance of an individual familiar with the child’s intake, whereas children aged ≥12 y answer the questions for themselves. Participants who complete the dietary component of NHANES are assigned specialized sampling weights to account for nonresponse and the day of the week of recall (ie, weekday compared with weekend). Dietary recall data are imported into *Survey Net*, a USDA food coding and data management system [31]. The USDA’s Food and Nutrient Database for Dietary Studies, which includes comprehensive food composition information on individual foods and beverages in the United States food system, is used to translate dietary intake data into specific nutrient intakes [32].

### Analytic sample and study variables

**FIGURE 1:**
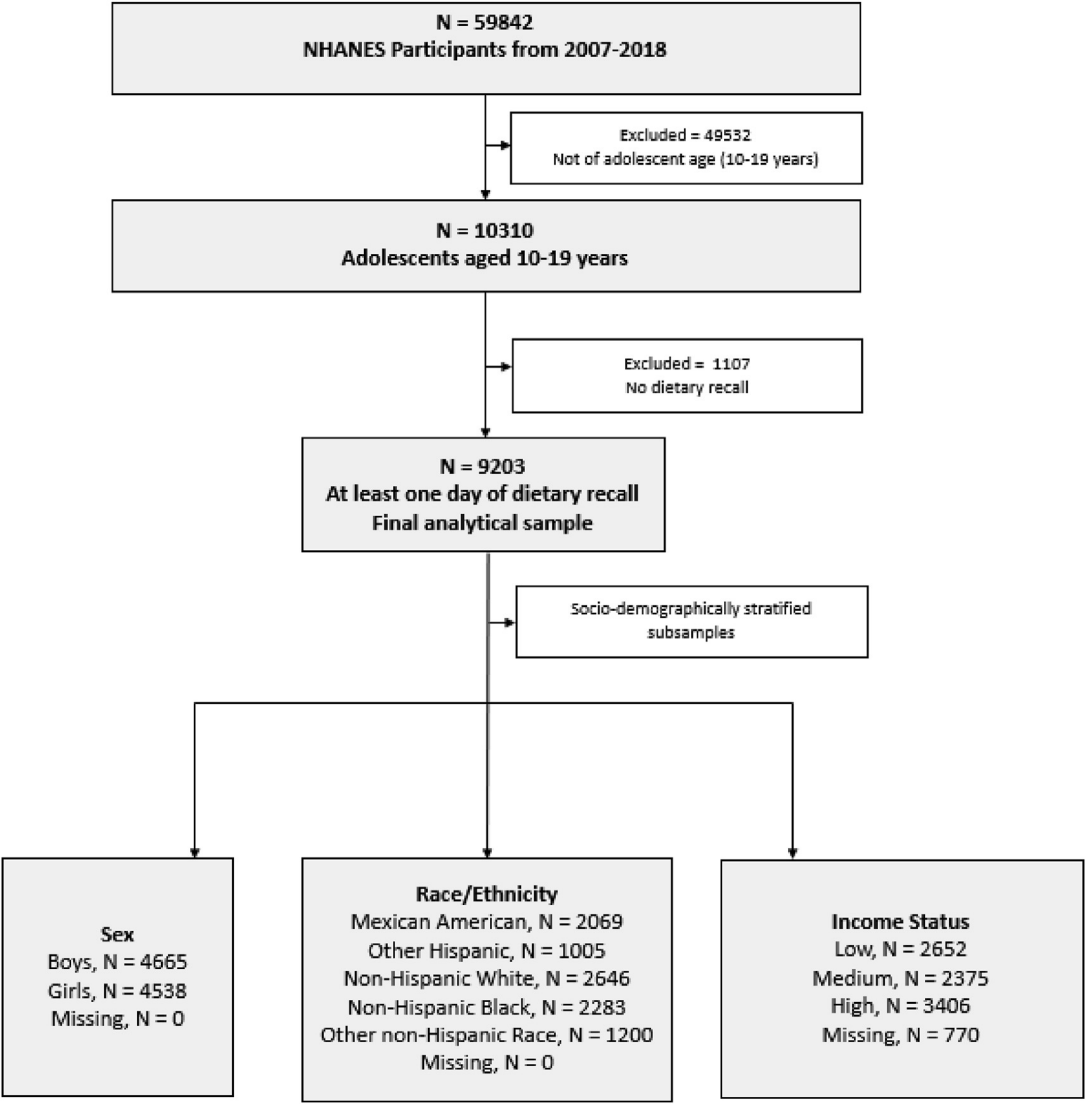
Participant flow chart.

Our eligible sample consisted of 10,310 adolescent boys and girls aged 10–19 y, 9203 of whom had ≤1 d of dietary recall data (Figure 1). The elements of NHANES data chosen for this study were 2 d of 24-h dietary recall and demographic information. In addition to dietary data, the variables chosen for this study include sex, race/ethnicity, and income status. Beginning with the 2011–2012 cycle, non-Hispanic Asian persons were oversampled, which made producing estimates for this group possible; however, to provide consistent race/ethnicity categories across the 6 cycles of data used, separate estimates for non-Hispanic Asian persons were not calculated. The continuous variable for the ratio of family income to poverty (0–5), based on the Department of Health and Human Services’ poverty guidelines, was used to construct a categorical variable for income status, where low is <1, medium is between 1 and 2, and high is >2 [33].

### Statistical analyses

All statistical procedures accounted for the complex, multistage, probability sampling design used in NHANES, where appropriate. To facilitate pooled analyses of all 6 continuous NHANES cycles, sampling weights (ie, the 2-y sample weights and specialized dietary weights for each day of data collection: day 1 or 2-d sample weight) for each 2-y cycle were combined using a fixed fraction (1/6) of the constituent surveys as per NCHS analytic guidelines [34]. Participant characteristics were summarized using descriptive statistics. Continuous variables are presented as mean ± unweighted SD or weighted mean ± weighted SE, and categorical variables are presented as unweighted counts with weighted percentages and CIs (95% CI) to provide nationally representative estimates.

To estimate adolescent food group consumption, we used the USDA’s Food Patterns Equivalents Database (FPED), which converts the foods from the Food and Nutrient Database for Dietary Studies into distinct food pattern components [35]. Then, we recategorized 13 FPED groups and individual foods as needed to match the 10 food groups included in the MDD-W indicator (Table 1), counting food groups only when the quantity consumed was ≥15 g, in keeping with standard practice for this indicator [4,6,28]. Food group consumption was estimated as the prevalence of intake of each of 10 food groups on each day of dietary recall. Then, a composite metric of the number of food groups consumed on a given day, which we termed adolescent dietary diversity score (ADDS), was derived for each adolescent, where 1 point was awarded per food group consumed for a maximum score of 10. ADDS was estimated for the entire sample and by sociodemographic category, in addition to the distribution of scores from 1 to 10, estimated as the proportion of adolescents with each of 10 intake scores. The few adolescents who consumed 0 food groups (based on the classification of dietary intake data using the 15-g cutoff [28]) were excluded from the analysis of ADDS (*n* = 5). The use of a numeric ADDS enabled us to quantify the extent of diversity of food groups consumed by the entire sample and by sociodemographic category.

**TABLE 1:** Food groups from the United States Department of Agriculture Food Patterns Equivalents Database recategorized to match minimum dietary diversity for women food groups

Food Patterns Equivalents Database	Minimum dietary diversity for women (MDD-W)
Total grains	Grains, white roots and tubers, and plantains
Potatoes	
Other starchy vegetables	
Beans, peas, and lentils	Pulses (beans, peas, or lentils)
Soy products	
Nuts and seeds	Nuts and seeds
Total dairy	Milk and milk products
Total meat, poultry, and seafood	Meat, poultry, and fish
Eggs	Eggs
Dark green vegetables	Dark green leafy vegetables
Total red and orange vegetables	Vitamin A–rich fruits and vegetables
Other vegetables	Other vegetables
Total fruit^1^	Other fruits

1 Orange fruits were removed from the total fruit category and added to the new category vitamin A–rich fruits and vegetables.

Differences in the proportion of adolescents consuming specific food groups between 2 d of dietary recall and by predefined sociodemographic categories, such as sex, race/ethnicity, and income status, were tested using the Rao–Scott χ^2^ tests. After initial omnibus testing, no multiple comparison tests were performed; rather, in post hoc analyses, 2-way significance tests with a reference category were performed for all pairwise hypotheses. Comparisons of proportions of food groups consumed were expressed as population prevalence differences (in percentage points) and prevalence ratios (PR with 95% CI). All analyses were performed using SAS version 9.4, and statistical significance was set at a 2-sided α level of 0.05.

## Results

### Description of the sample

A total eligible sample size of 10,310 was obtained from the combined 6 cycles of NHANES data (Table 2), 9203 of whom had at least the first day of dietary recall, indicating a participation rate of 89% on day 1 of 24-h dietary recall. Among these, 7934 adolescents had 2 days of dietary recall, resulting in a participation rate of 86% on day 2 of 24-h dietary recall. The mean age was 14.2 (± 2.9) y and the ratio of boys to girls was nearly 1:1 in all cycles, with the greatest difference in the 2007–2008 cycle (51.6% boys compared with 48.4% girls). The total sample consisted of 55.2% non-Hispanic White adolescents with the next largest groups being Mexican American adolescents at 14.4% and non-Hispanic Black adolescents at 14.3%. A comparison of the eligible population of 10,310 adolescents aged 10–19 y with those who had 1 day of dietary recall data (ie, the analytic subpopulation of 9203) indicated no differences in age, race/ethnicity, or household income status, suggesting no differential, and/or selection biases (results not shown). A secondary comparison of the population of 1107 who did not have dietary data to the analytic subpopulation of 9203 demonstrated that, overall, those without dietary data were slightly younger, more likely to be from high-income families, and more likely to be of other Hispanic or other non-Hispanic race (results not shown).

**TABLE 2:** Characteristics of adolescents aged 10–19 y in the NHANES, 2007–2018^1^

NHANES data collection cycle	2007–2008	2009–2010	2011–2012	2013–2014	2015–2016	2017–2018	Total
Sample size	1664	1758	1687	1878	1741	1582	10,310
Age, mean ± SD	14.2 ± 2.9	14.3 ± 2.9	14.2 ± 2.9	14.2 ± 2.9	14.1 ± 2.8	14.2 ± 2.9	14.2 ± 2.9
Sex, n (%)							
Boys	867 (51.6)	914 (50.2)	847 (50.0)	932 (50.3)	879 (51.0)	779 (50.0)	5218 (50.5)
Girls	797 (48.4)	844 (49.8)	840 (50.0)	946 (49.7)	862 (49.0)	803 (50.0)	5092 (49.5)
Race/ethnicity, n (%)							
Mexican American	391 (11.7)	489 (14.2)	296 (14.2)	435 (15.7)	377 (14.4)	296 (16.6)	2284 (14.4)
Other Hispanic	231 (6.6)	197 (5.8)	192 (7.7)	191 (6.9)	216 (8.3)	123 (7.9)	1150 (7.2)
Non-Hispanic White	525 (60.9)	573 (58.0)	382 (55.0)	487 (54.1)	477 (53.4)	477 (50.1)	2921 (55.2)
Non-Hispanic Black	439 (14.9)	387 (14.5)	512 (15.3)	468 (14.0)	386 (13.8)	360 (13.4)	2552 (14.3)
Other non-Hispanic^2^	78 (5.9)	112 (7.6)	305 (8.0)	297 (9.3)	285 (10.1)	326 (11.9)	1403 (8.8)
Income status, n (%)^3^							
Low	469 (22.8)	514 (22.3)	524 (24.8)	631 (25.4)	430 (18.2)	380 (22.6)	2948 (22.7)
Medium	409 (22.3)	445 (21.7)	409 (23.5)	434 (22.4)	483 (25.1)	413 (24.4)	2593 (23.3)
High	643 (54.9)	634 (55.9)	602 51.7)	675 (52.2)	654 (56.7)	604 (52.9)	3812 (54.1)

1 Values are unweighted counts and weighted percentages except where otherwise indicated.

2 The other non-Hispanic category included multiracial, Asian, and other race/ethnicity.

3 The continuous variable for the ratio of family income to poverty (0–5) was used to construct a categorical variable for income status, where low is <1, medium is between 1 and 2, and high is >2. Missing data for income status resulted in lower total counts for this variable for each cycle and for the overall sample.

### Food group consumption

**FIGURE 2:**
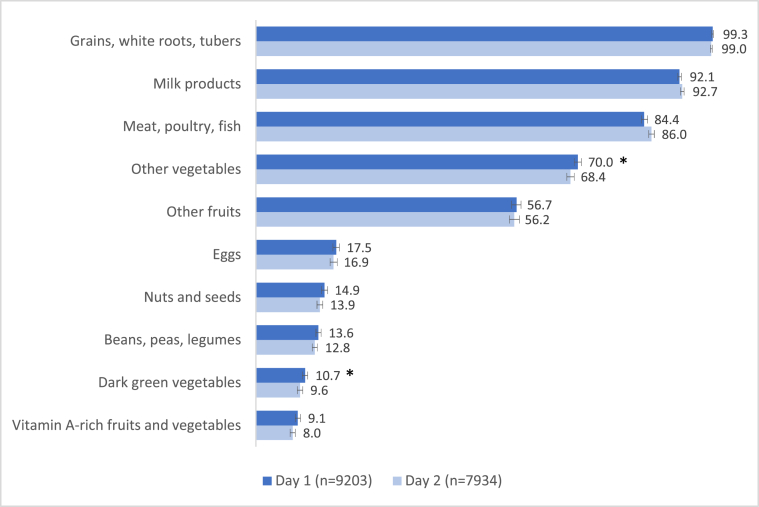
Differences in proportion (%) of adolescents aged 10–19 y consuming each of the 10 food groups between 2 d of 24-h dietary recall in the NHANES, 2007–2018 (*n* = 9203). ∗Significant difference in proportion consuming food groups between day 1 and day 2 of dietary recall based on Rao-Scot χ^2^, *P* < 0.05.

Across all food groups, there was little variation in the frequency of consumption between days 1 and 2 of 24-h dietary recall (Figure 2). Starchy foods and animal products were the most commonly consumed at >80% on both days across 3 food groups: grains, white roots, and tubers (99.0% and 99.3%), milk products (92.1% and 92.7%), and meat, poultry, and fish (84.4% and 86.0%). Conversely, <15% of adolescents consumed key micronutrient-dense foods, such as vitamin A–rich fruits and vegetables (8.0% and 9.1%), dark green vegetables (9.6% and 10.7%), beans, peas, and legumes (12.8% and 13.6%), and nuts and seeds (13.9% and 14.9%) over the 2 d of recall. The only significant differences in food group consumption across the 2 d of recall were for other vegetables (1.6 percentage points different; *P* = 0.001) and dark green vegetables (1.1 percentage points different, *P* = 0.038). Due to the similarity in consumption patterns and minimal differences in prevalence (<2%) of food group intake, all subsequent analyses and conclusions of this work were based on the first day of dietary recall.

Disaggregating consumption by sex, boys were 28% more likely to consume eggs (PR: 1.28; *P* = 0.001) (Table 3), 7% more likely to consume meat, poultry, and fish (PR: 1.07; *P* < 0.0001), and 26% less likely to consume vitamin A–rich fruits and vegetables (PR: 0.74; *P* = 0.001) on a given day than girls. Other significant differences by sex were small in magnitude (ie, ≤5%), and there was no significant difference in the consumption of grains, white, roots and tubers, beans, peas, and legumes, nuts and seeds, dark green vegetables, or other fruit between boys and girls.

**TABLE 3:** Differences in proportion (%) of adolescents aged 10–19 y consuming each of the 10 food groups on day 1 of dietary recall by sex, race/ethnicity, and income status in the NHANES, 2007–2018 (*n* = 9203)

Food group	Unweighted *n*	% (CI)^3^	*P* value for differences across strata^4^	Prevalence difference (PD, 95% CI)	Prevalence ratio (PR, 95% CI)	*P* value^5^
**Grains,white,roots, and tubers**						
*All adolescents*	9128	99.3 (99.1–99.5)				
Sex			0.295			
Boys	4640	99.4 (99.1–99.7)		+0.23 (−0.19, +0.64)	1.00 (1.00,1.01)	0.295
Girls	4488	99.2 (98.9–99.5)		Ref	Ref	Ref
Race/ethnicity			0.347			
Mexican American	2050	98.9 (98.3–99.4)		−0.58 (−1.22, +0.06)	0.99 (0.99, 1.00)	0.046
Other Hispanic	996	99.3 (98.7–99.9)		−0.13 (−0.78, +0.53)	1.00 (0.99, 1.01)	0.691
Non-Hispanic Black	2266	99.2 (98.7–99.7)		−0.23 (−0.76, +0.30)	1.00 (0.99, 1.00)	0.366
Other non-Hispanic^1^	1190	99.2 (98.4–100.0)		−0.26 (−1.06, +0.55)	1.00 (0.99, 1.01)	0.475
Non-Hispanic White	2626	99.4 (99.1–99.8)		Ref	Ref	Ref
Income status^2^			0.039			
Low	2627	99.1 (98.5–99.6)		−0.50 (−1.10, +0.09)	0.99 (0.99, 1.00)	0.053
Medium	2352	99.0 (98.4–99.5)		−0.61 (−1.17, −0.04)	0.99 (0.99, 1.00)	0.017
High	3385	99.6 (99.3–99.8)		Ref	Ref	Ref
**Beans, peas, and legumes**						
*All adolescents*	1374	13.6 (12.5–14.7)				
Sex			0.257			
Boys	714	13.1 (11.5–14.6)		−1.01 (−2.78, +0.75)	0.93 (0.81, 1.06)	0.257
Girls	660	14.1 (12.8–15.3)		Ref	Ref	Ref
Race/ethnicity			<0.0001			
Mexican American	446	22.1(19.4–24.8)		+11.02 (+7.90, +14.14)	1.99 (1.66, 2.40)	<0.0001
Other Hispanic	211	19.2 (15.1–23.3)		+8.10 (+3.59, +12.61)	1.73 (1.33, 2.26)	<0.0001
Non-Hispanic Black	240	10.6 (9.0–12.2)		−0.51 (−2.67, +1.66)	0.95 (0.78, 1.17)	0.642
Other non-Hispanic^1^	184	15.0 (10.9–19.2)		+3.94 (−0.32, +8.20)	1.36 (1.01, 1.82)	0.042
Non-Hispanic White	293	11.1 (9.6–12.6)		Ref	Ref	Ref
Income status^2^			0.048			
Low	417	14.8 (13–16.5)		+2.47 (−0.20, +5.14)	1.20 (0.98, 1.47)	0.066
Medium	369	15.6 (12.9–18.2)		+3.28 (−0.10, +6.66)	1.27 (1, 1.61)	0.048
High	463	12.3 (10.5–14)		Ref	Ref	Ref
**Nuts and seeds**						
*All adolescents*	1149	14.9 (13.5–16.2)				
Sex			0.711			
Boys	606	15.1 (13.3–16.9)		+0.42 (−1.82, +2.65)	1.03 (0.88, 1.20)	0.711
Girls	543	14.7 (12.9–16.4)		Ref	Ref	Ref
Race/ethnicity			<0.0001			
Mexican American	236	12.1 (9.9–14.4)		−4.96 (−7.98, −1.95)	0.71 (0.57, 0.88)	0.001
Other Hispanic	91	10.9 (7.6–14.1)		−6.23 (−10.24, −2.22)	0.64 (0.46, 0.89)	0.004
Non-Hispanic Black	247	10.8 (8.7–12.8)		−6.32 (−8.97, −3.68)	0.63 (0.51, 0.77)	<0.0001
Other non-Hispanic^1^	169	15.7 (12.2–19.3)		−1.34 (−5.55, +2.87)	0.92 (0.71, 1.20)	0.533
Non-Hispanic White	406	17.1 (15.0–19.2)		Ref	Ref	Ref
Income status^2^			0.0003			
Low	276	11.8 (9.8–13.7)		−5.49 (−8.93, −2.05)	0.68 (0.54, 0.87)	0.001
Medium	274	12.9 (10.7–15)		−4.37 (−7.31, −1.43)	0.75 (0.61, 0.91)	0.003
High	517	17.2 (14.9–19.6)		Ref	Ref	Ref
**Milk products**						
*All adolescents*	8381	92.1 (91.4–92.9)				
Sex			0.001			
Boys	4309	93.5 (92.5–94.4)		+2.64 (+1.12, +4.17)	1.03 (1.01,1.05)	0.001
Girls	4072	90.8 (89.6–92)		Ref	Ref	Ref
Race/ethnicity			<0.0001			
Mexican American	1921	93.2 (92.1–94.3)		+0.16 (−1.49, +1.81)	1.00 (0.98, 1.02)	0.848
Other Hispanic	927	92.4 (90.3–94.5)		−0.62 (−3.05, +1.81)	0.99 (0.97, 1.02)	0.603
Non-Hispanic Black	2016	88.6 (87.0–90.2)		−4.43 (−6.39, −2.48)	0.95 (0.93, 0.97)	<0.0001
Other non-Hispanic^1^	1059	90.3 (88.3–92.3)		−2.72 (−4.96, −0.48)	0.97 (0.95, 0.99)	0.010
Non-Hispanic White	2458	93.0 (91.8–94.2)		Ref	Ref	Ref
Income status^2^			0.453			
Low	2386	91.7 (90.1–93.2)		−1.04 (−2.97, +0.88)	0.99 (0.97, 1.01)	0.279
Medium	2152	91.9 (90.5–93.4)		−0.80 (−2.63, +1.03)	0.99 (0.97, 1.01)	0.382
High	3154	92.7 (91.5–94)		Ref	Ref	Ref
**Meat, poultry, and fish**						
*All adolescents*	7903	84.4 (82.9–85.9)				
Sex			<0.0001			
Boys	4101	87.4 (85.9–88.8)		+5.92 (+3.33, +8.51)	1.07 (1.04, 1.11)	<0.0001
Girls	3802	81.4 (79–83.9)		Ref	Ref	Ref
Race/ethnicity			0.007			
Mexican American	1779	86.1 (83.9–88.4)		+3.01 (−0.65, +6.67)	1.04 (0.99, 1.08)	0.098
Other Hispanic	861	84.9 (82.2–87.7)		+1.82 (−1.93, +5.56)	1.02 (0.98, 1.07)	0.342
Non-Hispanic Black	2030	88.3 (86.3–90.2)		+5.18 (+2.00, +8.35)	1.06 (1.02, 1.10)	0.001
Other non-Hispanic^1^	997	82.6 (78.9–86.2)		−0.54 (−5.05, +3.98)	0.99 (0.94, 1.05)	0.813
Non-Hispanic White	2236	83.1 (80.5–85.7)		Ref	Ref	Ref
Income status^2^			0.109			
Low	2302	86.4 (84.2–88.6)		+3.08 (−0.08, +6.24)	1.04 (1.00, 1.08)	0.049
Medium	2023	84.9 (82.9–86.9)		+1.63 (−1.49, +4.74)	1.02 (0.98, 1.06)	0.294
High	2913	83.3 (80.8–85.8)		Ref	Ref	Ref
**Eggs**						
*All adolescents*	1643	17.5 (16.3–18.8)				
Sex			0.001			
Boys	889	19.7 (17.8–21.6)		+4.36 (+1.75, +6.96)	1.28 (1.11, 1.49)	0.001
Girls	754	15.3 (13.6–17)		Ref	Ref	Ref
Race/ethnicity			0.002			
Mexican American	426	20.7 (18.2–23.2)		+4.11 (+0.84, +7.38)	1.25 (1.05, 1.49)	0.015
Other Hispanic	212	19.5 (16.5–22.5)		+2.91 (−0.74, +6.57)	1.18 (0.96, 1.43)	0.110
Non-Hispanic Black	336	14.0 (11.8–16.2)		−2.62 (−5.67, +0.43)	0.84 (0.69, 1.03)	0.084
Other non-Hispanic^1^	237	21.8 (16.9–26.6)		+5.14 (−0.45, +10.74)	1.31 (1.00, 1.72)	0.057
Non-Hispanic White	432	16.6 (14.5–18.8)		Ref	Ref	Ref
Income status^2^			0.438			
Low	492	18.3 (15.9–20.6)		+1.61 (−1.27, +4.49)	1.10 (0.93, 1.29)	0.263
Medium	417	18.0 (15.8–20.3)		+1.36 (−1.57, +4.3)	1.08 (0.91, 1.28)	0.352
High	600	16.7 (14.8–18.5)		Ref	Ref	Ref
**Dark green vegetables**						
*All adolescents*	939	10.7 (9.5–12.0)				
Sex			0.192			
Boys	455	10.0 (8.4–11.7)		−1.43 (−3.6, +0.74)	0.87 (0.71, 1.07)	0.192
Girls	484	11.5 (9.8–13.1)		Ref	Ref	Ref
Race/ethnicity			<0.0001			
Mexican American	156	7.7 (6.0–9.3)		−3.30 (−5.95, −0.65)	0.70 (0.53, 0.93)	0.010
Other Hispanic	88	9.7 (6.9–12.5)		−1.25 (−4.83, +2.33)	0.89 (0.62, 1.26)	0.494
Non-Hispanic Black	213	9.9 (8.1–11.7)		−1.09 (−3.61, +1.43)	0.90 (0.71, 1.15)	0.385
Other non-Hispanic^1^	234	16.8 (13.7–19.9)		+5.79 (+1.74, +9.84)	1.53 (1.14, 2.04)	0.004
Non-Hispanic White	248	11.0 (8.9–13.1)		Ref	Ref	Ref
Income status^2^			0.031			
Low	232	9.7 (7.7–11.7)		−2.51 (−5.03, +0.01)	0.79 (0.63, 1.01)	0.049
Medium	217	9.5 (7.7–11.4)		−2.68 (−5.12, −0.23)	0.78 (0.62, 0.98)	0.031
High	413	12.2 (10.3–14.1)		Ref	Ref	Ref
**Vitamin A–rich fruits and veg**						
*All adolescents*	785	9.1 (7.9–10.3)				
Sex			0.001			
Boys	357	7.7 (6.5–9)		−2.71 (−4.28, -1.14)	0.74 (0.62, 0.88)	0.001
Girls	428	10.5 (8.9–12)		Ref	Ref	Ref
Race/ethnicity			0.001			
Mexican American	177	8.2 (6.4–9.9)		−1.87 (−4.31, +0.57)	0.81 (0.62, 1.07)	0.119
Other Hispanic	91	9.4 (7–11.8)		−0.66 (−3.41, +2.10)	0.93 (0.70, 1.25)	0.639
Non-Hispanic Black	133	5.4 (4.2–6.6)		−4.64 (−6.79, −2.49)	0.54 (0.41, 0.71)	<0.0001
Other non-Hispanic^1^	131	10.6 (7.4–13.8)		+0.55 (−2.94, +4.04)	1.05 (0.76, 1.47)	0.752
Non-Hispanic White	253	10.0 (8.3–11.8)		Ref	Ref	Ref
Income status^2^			0.210			
Low	220	8.2 (6.3–10.2)		−1.81 (−4.04, +0.42)	0.82 (0.64, 1.06)	0.114
Medium	183	8.4 (6.5–10.4)		−1.59 (−3.98, +0.80)	0.84 (0.64, 1.10)	0.195
High	328	10.0 (8.4–11.6)		Ref	Ref	Ref
**Other veg**						
*All adolescents*	6412	70.0 (68.5–71.5)				
Sex			0.004			
Boys	3342	71.9 (69.8–73.9)		+3.62 (+1.15, +6.08)	1.05 (1.02, 1.09)	0.004
Girls	3070	68.2 (66.4–70.1)		Ref	Ref	Ref
Race/ethnicity			0.001			
Mexican American	1531	73.9 (71.3–76.4)		+3.25 (+0.05, +6.45)	1.05 (1.00, 1.09)	0.043
Other Hispanic	701	67.9 (63.6–72.2)		−2.73 (−7.57, +2.10)	0.96 (0.90, 1.03)	0.257
Non-Hispanic Black	1495	64.8 (62.0–67.7)		−5.79 (−9.51, −2.07)	0.92 (0.87, 0.97)	0.002
Other non-Hispanic^1^	849	70.2 (66.4–74.1)		−0.37 (−4.36, +3.61)	0.99 (0.94, 1.05)	0.852
Non-Hispanic White	1836	70.6 (68.4–72.8)		Ref	Ref	Ref
Income status^2^			0.653			
Low	1815	70.4 (67.8–73)		+0.19 (−3.42, +3.80)	1.00 (0.95, 1.06)	0.917
Medium	1629	68.8 (65.8–71.7)		−1.47 (−5.13, +2.20)	0.98 (0.93, 1.03)	0.427
High	2421	70.2 (67.9–72.6)		Ref	Ref	Ref
**Other fruit**						
*All adolescents*	5274	56.7 (54.8–58.5)				
Sex			0.349			
Boys	2652	56.0 (53.5–58.5)		−1.36 (−4.24, +1.53)	0.98 (0.93, 1.03)	0.349
Girls	2622	57.4 (55.1–59.6)		Ref	Ref	Ref
Race/ethnicity			<0.0001			
Mexican American	1266	63.2 (60.4–65.9)		+9.32 (+5.64, +12.99)	1.17 (1.10, 1.25)	<0.0001
Other Hispanic	620	60.1 (55.9–64.4)		+6.27 (+0.53, +12.01)	1.12 (1.01, 1.23)	0.031
Non-Hispanic Black	1310	57.2 (54.4–60.1)		+3.34 (−1.06, +7.74)	1.06 (0.98, 1.15)	0.128
Other non-Hispanic^1^	705	59.4 (55.2–63.7)		+5.54 (+0.38, +10.70)	1.10 (1.01, 1.21)	0.034
Non-Hispanic White	1373	53.9 (50.8–57)		Ref	Ref	Ref
Income status^2^			0.003			
Low	1498	54.9 (52–57.8)		−4.11 (−8.45, 0.23)	0.93 (0.86, 1)	0.059
Medium	1292	52.2 (49.1–55.3)		−6.80 (−11.10, −2.51)	0.88 (0.82, 0.96)	0.001
High	2026	59.0 (56–62.1)		Ref	Ref	Ref

1 The other non-Hispanic category included multiracial, Asian, and other race/ethnicity.

2 The continuous variable for the ratio of family income to poverty (0–5) was used to construct a categorical variable for income status, where low is <1, medium is between 1 and 2, and high is >2. Missing data for income status resulted in lower total counts for this variable.

3 All percentage values have been weighted to reflect national estimates.

4 *P* values for differences in prevalence within each strata were estimated using the Rao–Scott χ^2^ test.

5 *P* values for pairwise prevalence differences and prevalence ratios were estimated using the Rao–Scott χ^2^ test.

Differences in consumption by race/ethnicity were significant for all food groups except grains, white, roots, and tubers (Table 3). Egg consumption was the highest among Mexican American adolescents, who were 25% more likely to consume eggs (PR: 1.25; *P* = 0.015) than non-Hispanic White adolescents on a given day. Compared with non-Hispanic White adolescents, Mexican American and other Hispanic adolescents were 99% (PR: 1.99; *P* < 0.0001) and 73% (PR: 1.73; *P* < 0.0001) more likely to consume beans, peas, and legumes, respectively. Consumption of nuts and seeds was the highest among non-Hispanic White adolescents, who were 36%–37% more likely to consume this food group than their other Hispanic (PR: 0.64; *P* = 0.004) and non-Hispanic Black (PR: 0.63; *P* < 0.0001) peers. Compared with their non-Hispanic White peers, adolescents of other non-Hispanic race were 53% more likely to consume dark green vegetables (PR: 1.53; *P* = 0.004), whereas Mexican American adolescents were 30% less likely to do so (PR: 0.70; *P* = 0.010). Mexican American adolescents were 5% more likely to consume other vegetables (PR: 1.05; *P* = 0.043) than their non-Hispanic White peers, whereas non-Hispanic Black adolescents were 8% less likely to do so (PR: 0.92; *P* = 0.002). Moreover, non-Hispanic Black adolescents were 46% less likely to consume vitamin A–rich fruits and vegetables than non-Hispanic White adolescents (PR: 0.54; *P* < 0.0001). Consumption of other fruit was the highest among Mexican American adolescents and other Hispanic adolescents, who were 17% more likely (PR: 1.17; *P* < 0.0001) and 12% more likely (PR: 1.12; *P* = 0.031) to consume this food group than their non-Hispanic White peers, respectively.

There were statistically significant differences in the consumption of 5 of the 10 food groups by income status: grains, white, roots, and tubers; beans, peas, and legumes; nuts and seeds; dark green vegetables; and other fruit (Table 3). The most notable difference was for nuts and seeds; adolescents from low-income families were 32% less likely to consume nuts and seeds than their peers from high-income families (PR: 0.68; *P* = 0.001). Conversely, consumption of beans, peas, and legumes was 27% higher (PR: 1.27; *P* = 0.048) among adolescents from medium-income families compared to high-income families. Adolescents from low-income and medium-income families were 21%–22% less likely to consume dark green vegetables than their peers from wealthier families (PR: 0.79; *P* = 0.049; PR: 0.78; *P* = 0.031, respectively). Adolescents from medium-income families were also 12% less likely to consume other fruit than those from high-income families (PR: 0.88; *P* = 0.001).

### Adolescent dietary diversity score

**FIGURE 3:**
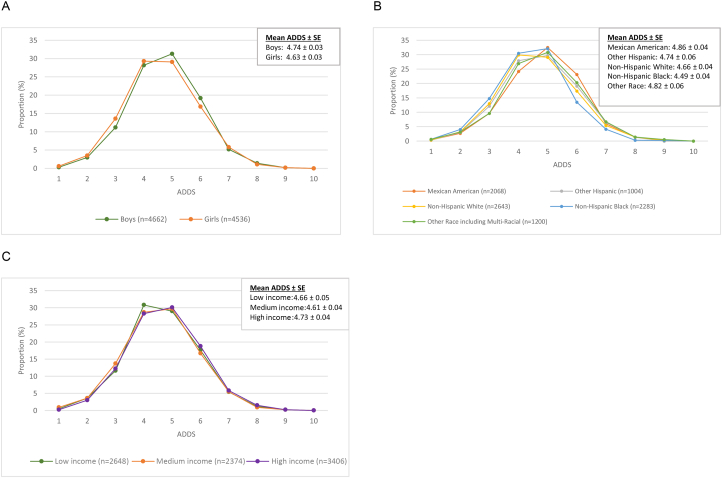
Proportion (%) of adolescents aged 10–19 y by adolescent dietary diversity score (ADDS) on day 1 of dietary recall in the NHANES, 2007–2018 (*n* = 9198). ADDS is a composite metric where 1 point is awarded per food group consumed for a maximum score of 10. Five adolescents who consumed 0 food groups were excluded from the analysis of ADDS. Panels show various strata examined: (A) sex; (B) race/ethnicity; and (C) income status.

The overall mean ADDS was 4.69. The mean ADDS of boys and girls was 4.74 and 4.63, respectively (Figure 3). Across race and ethnic groups, the mean ADDS ranged from 4.49 among non-Hispanic Black adolescents to 4.86 among Mexican American adolescents. By income status, the mean ADDS was the lowest among adolescents of medium-income status (4.61) and the highest among adolescents of high-income status (4.73). The distributions of ADDS by sex, race/ethnicity, and income status demonstrate that, across all strata, adolescents tended to consume 4–5 food groups, with substantially fewer consuming 6 or more.

## Discussion

For the entire study population, and across all demographic characteristics, the mean ADDS was <5 and ranged by <1 food group. The MDD-W indicator, from which the 10 food groups examined in this study were chosen, has been validated among women of reproductive age (aged 15–49 y) with a minimum of 5 food groups as a proxy for higher micronutrient adequacy [6,28]. Although no cutoff has yet been established for adolescents aged 10–19 y, our finding of a mean population score of <5 suggests that the diets of adolescents in the United States may put them at risk of micronutrient deficiency.

Multiple studies that have sought to quantify adolescent dietary diversity across geographic contexts have found similar population scores to that found in this study. A 2018 study of pregnant adolescent girls in Bangladesh found a mean dietary diversity score of 5.27 [7], whereas a 2022 study in India found a mean dietary diversity score of 4.5 among adolescent girls [36]. A 2020 study found a mean dietary diversity score of 4.61 among older adolescents across 8 Latin-American countries [8]. Two recent studies in geographically distant regions of Ethiopia found a mean dietary diversity score of 4.9 among high school girls [37] and a mean dietary diversity score of 4.73 among adolescent boys and girls [38]. Moreover, a 2017 multicountry study found mean dietary diversity scores ranging from 3.52 in Ethiopia to 5.34 in Peru [15]. Although the measures used across these studies are not identical, the ranges of these measures are similar because all studies used women’s dietary diversity score, MDD-W, or individual dietary diversity score. Overall, the findings suggest similarity in dietary diversity score among adolescents from diverse populations, with scores generally not surpassing 5 in most contexts. A strong positive bivariate relationship between the number of food groups consumed and mean probability of adequacy has been established in the literature [4], and researchers have concluded that variety in the diet increases the probability of achieving micronutrient adequacy [3]. Thus, our findings indicate that, although United States adolescents may have a higher consumption of certain food groups (eg, starchy foods and animal products), their overall dietary diversity is not superior to that of their peers in less-affluent countries and suggests a lack of variety, which may pose a risk for meeting nutrient needs.

We found few differences in food group consumption patterns across days 1 and 2 of dietary recall, but substantial variation in the proportion of adolescents consuming each of 10 food groups and variation in consumption patterns across sociodemographic categories. Prevalence of consumption of starchy foods and animal products (ie, grains, white roots, and tubers; milk products; and meat, poultry, and fish) was high overall and across all characteristics. The high prevalence of consumption of these food groups is consistent with a Western dietary pattern, which has been identified as a key contributing factor to the rise in obesity and other chronic diseases [[39], [40], [41]]. In addition, the relatively low prevalence of consumption of several micronutrient-rich food groups (ie, vitamin A–rich fruits and vegetables; dark green vegetables; beans, peas, and legumes; and nuts and seeds) among United States adolescents in this study suggests a lower likelihood of achieving micronutrient adequacy [3,4,7] and is consistent with a recent study that found suboptimal diet quality in United States adolescents [26]. Our finding that more than half of adolescents consumed other fruits on both days of recall (Figure 2), and 70% consumed other vegetables, is encouraging; however, the positive nature of this finding should not be overestimated because it does not provide an indication of the number of servings or total amount of fruits and vegetables consumed. A recent study using data from the 2019 Behavioral Risk Factor Surveillance System found that, among American adults, only 12.3% met fruit recommendations and 10.0% met vegetable recommendations as set forth by the Dietary Guidelines for Americans [42].

Our finding that approximately only 1 in 10 American adolescents consumed dark green vegetables on either day of dietary recall is aligned with previous studies in American adults [43] and youth [26] and a study of Mexican youth and adults [44]. However, compared with Mexican adolescents living in Mexico, the Mexican American adolescents in our study had substantially lower consumption of vitamin A–rich fruits and vegetables, and lower consumption of beans, peas, legumes, and eggs. Conversely, Mexican adolescents living in Mexico showed lower consumption of meat, poultry, and fish; milk products; nuts and seeds; and other fruits than the Mexican American adolescents in our study. These findings suggest that, although the Western diet is increasingly common in Mexico [45], important differences persist in the dietary patterns of youth from these neighboring countries.

Beans, peas, and legumes were consumed by a higher proportion of Mexican American adolescents and other Hispanic adolescents than adolescents of non-Hispanic race/ethnicity, which is consistent with cultural preferences and traditional dietary patterns [46]. Recent research has shown that Latino families who have immigrated to the United States report serving mostly traditional foods at home [47], while another study found that Mexican children from immigrant households in the United States tend to eat more acculturated foods in schools and restaurants than that at home [48].

Consumption of nuts and seeds was most prevalent among non-Hispanic White adolescents and adolescents from high-income status households. This finding aligned with a study in United States adults, which found that more non-Hispanic White adults consumed nuts on a given day than non-Hispanic Black or Hispanic adults, suggesting that cultural preference and household food norms may play a role in consumption levels of this food group [49]. The differential consumption of nuts and seeds by income status may be linked to financial access to this food group, which is relatively more expensive than many of the other food groups. Previous research has shown that cost is a key barrier to consumption of nuts in multiple country contexts [50].

Although consumption of dark green vegetables and vitamin A–rich fruits and vegetables was low across all groups in our study, adolescents of high-income status consumed dark green vegetables more frequently than their lower-income peers on a given day. Dark green vegetable consumption was also substantially higher among adolescents of other non-Hispanic race than that among non-Hispanic White adolescents, whereas consumption of vitamin A–rich fruits and vegetables was the lowest among non-Hispanic Black adolescents. Although financial access is less likely to be a barrier for these particular food groups, these results could be related to a confluence of cultural preference, socioeconomic status, and neighborhood food environments. Research has shown that higher socioeconomic status is associated with higher consumption of produce, especially for those residing in lower poverty neighborhoods [51].

### Strengths and limitations

The strengths of our study include a large sample selected from 10 y of nationally representative data and the use of 2 days of 24-h dietary recall data and the USDA’s FPED to establish consumption patterns across 10 key food groups. Our combined survey sample size of 9203 is nationally representative of 41.7 million adolescents in the United States over the 10-y study period. Furthermore, the study had more than adequate statistical power to achieve the research objective. Despite these strengths, we recognized several limitations to this work. Beginning with the 2011–2012 NHANES data collection cycle, non-Hispanic Asian people were oversampled, allowing for stable estimates for this group; however, to ensure analytic consistency across the 6 cycles of data used, we did not calculate estimates for non-Hispanic Asian adolescents but rather included them within the other non-Hispanic race category. Furthermore, the 10 food groups chosen for this study are those established for the MDD-W indicator, which has been validated for use in women of reproductive age but not yet systematically tested for use in other groups. Therefore, we were not able to apply the cutoff of 5 food groups confidently to our study population of adolescents aged 10–19 y. The lack of a cutoff for the dichotomous indicator to serve as a proxy for achieving micronutrient adequacy in adolescents could be the subject of future research.

In conclusion, the mean ADDS among United States youth suggests an overall lack of variety. Our findings indicate that the dietary pattern exhibited by youth in the United States—characterized by relatively low prevalence of consumption of fruits, vegetables, nuts, and legumes—could pose a risk for adolescents’ ability to achieve micronutrient adequacy and carry implications for chronic disease risk later in life. It may be useful for policies and programs focusing on adolescent health and nutrition in the United States to incorporate targeted messaging on dietary diversity and, in particular, the benefits of consuming plant-based foods from multiple food groups. Differences in consumption patterns by sex, race/ethnicity, and income status could be used to inform further tailoring of nutrition programs and interventions.

## Author contributions

The authors’ responsibilities were as follows – MJJ, MEDJ, NJB, UR, RM, OYA: designed the research; MJJ, OYA: conducted the research, analyzed the data; and wrote the paper; MJJ: had primary responsibility for final content; and all authors: read and approved the final manuscript. The findings and conclusions in this report are those of the authors and do not necessarily represent the official position of the Centers for Disease Control and Prevention or of the Food and Agriculture Organization of the United Nations.

## Conflict of interest

The authors report no conflicts of interest.

### Funding

The authors reported no funding received for this study.

## Data availability

The data described in this manuscript are publicly and freely available without restriction at https://www.cdc.gov/nchs/nhanes/index.htm. Analytic code will be made available on request pending internal review and approval.
